# Hepatitis B & C epidemiology in Morocco

**DOI:** 10.1186/1753-6561-5-S1-P20

**Published:** 2011-01-10

**Authors:** Abdelouaheb Bennani, Warda Baha, Noureddine Dersi, M Mustapha Ennaji, Fatiha Lazaar, Abdelouahed El Malki, Mohammed Hassar

**Affiliations:** 1 Pasteur Institute of Morocco, Laboratoire de Biologie Moléculaire, Casablanca, Morocco; 2 Faculté des Sciences et Techniques, Mohammedia, Morocco

## 

Hepatitis B (HBV) and C (HCV) viruses are major public health problems worldwide and are a serious cause of liver disease that may silently progress toward cirrhosis and hepatocellular carcinoma.

The epidemiology of HBV and HCV infection in Morocco was studied. A large screening of HBsAg and HCVab was performed by third generation ELISA. Hepatitis B and C are parenterally transmitted with respective prevalence of about 1.79% and 1.5% of the general population studied. HCV RNA was detected in the fully automated Cobas Ampliprep/Cobas Amplicor V2.0 (Roche Diagnostics), HBV and HCV viral loads are measured by the CAP/CTM real-time PCR (Roche Diagnostics). HCV genotyping was tested by Versant LIPA HCV II (Siemens). We have shown that HCV genotypes 1 and 2 are the most prevalent in Morocco and HCV genotypes 3, 4 and 5 are were less common.

Sequencing of HCV NS5B region of 2a/2c and unclassified 2 genotype have permitted us to analyze 16 samples in order to confirm the accuracy of Inno-LiPa 5’NCR results, especially for 2a/2c genotype. Surprisingly, 15 samples were assigned to subtype 2i and one sample clustered with 2j/2k subtype. This finding suggests that subtype 2i is not only found in French patients as published, but also in Morocco with high prevalence.

In the other hand, HBV genotype D was predominant in our patients, as this is the major HBV genotype in Mediterranean countries. Furthermore, the wild type, mixed infection, BCP mutations and precore mutant were found in ten, thirty four, four and seven out of 55 HBV isolates, respectively. The A1762T/G1764A BCP dual mutation was not found in our isolates. Four samples presented single mutation in the BCP dual mutation region, whereas six showed a novel G1764T mutation.

In conclusion, HBV and HCV infections are prevalent in Morocco, HCV is parenterally transmitted and HBV is parenterally and also sexually transmitted in our country. Nosocomial transmission of those two viruses is important, especially in high risk groups (hemodialyzed patients & haemophiliacs). HBV genotype D predominates in Morocco as in Mediterranean countries, HBV genotypes A and F are quite rare; these are possibly acquired from other countries. High circulation of precore and basal core promoter mutants is common in chronic hepatitis B infection in Morocco.

